# A rapid qualitative assessment of oral cholera vaccine anticipated acceptability in a context of resistance towards cholera intervention in Nampula, Mozambique

**DOI:** 10.1016/j.vaccine.2017.10.087

**Published:** 2018-10-22

**Authors:** Rachel Démolis, Carlos Botão, Léonard W. Heyerdahl, Bradford D. Gessner, Philippe Cavailler, Celestino Sinai, Amílcar Magaço, Jean-Bernard Le Gargasson, Martin Mengel, Elise Guillermet

**Affiliations:** a Agence de Médecine Préventive, Bât. JB Say, 4e étage, aile A, 13 chemin du Levant, 01210 Ferney-Voltaire, France; b Instituto Nacional de Saúde, Avenido Eduardo Mundlane/Salvador Allende, Maputo, Mozambique; c Agence de Médecine Préventive, Abidjan, Cote d’Ivoire

**Keywords:** Anthropology, Cholera vaccines, Public health, Attitude to health, Politics, Mozambique

## Abstract

**Introduction:**

While planning an immunization campaign in settings where public health interventions are subject to politically motivated resistance, designing context-based social mobilization strategies is critical to ensure community acceptability. In preparation for an Oral Cholera Vaccine campaign implemented in Nampula, Mozambique, in November 2016, we assessed potential barriers and levers for vaccine acceptability.

**Methods:**

Questionnaires, in-depth interviews, and focus group discussions, as well as observations, were conducted before the campaign. The participants included central and district level government informants (national immunization program, logistics officers, public health directors, and others), community leaders and representatives, and community members.

**Results:**

During previous well chlorination interventions, some government representatives and health agents were attacked, because they were believed to be responsible for spreading cholera instead of purifying the wells. Politically motivated resistance to cholera interventions resurfaced when an OCV campaign was considered. Respondents also reported vaccine hesitancy related to experiences of problems during school-based vaccine introduction, rumors related to vaccine safety, and negative experiences following routine childhood immunization. Despite major suspicions associated with the OCV campaign, respondents’ perceived vulnerability to cholera and its perceived severity seem to override potential anticipated OCV vaccine hesitancy.

**Discussion:**

Potential hesitancy towards the OCV campaign is grounded in global insecurity, social disequilibrium, and perceived institutional negligence, which reinforces a representation of estrangement from the central government, triggering suspicions on its intentions in implementing the OCV campaign. Recommendations include a strong involvement of community leaders, which is important for successful social mobilization; representatives of different political parties should be equally involved in social mobilization efforts, before and during campaigns; and public health officials should promote other planned interventions to mitigate the lack of trust associated with perceived institutional negligence. Successful past initiatives include public intake of purified water or newly introduced medication by social mobilizers, teachers or credible leaders.

## Highlights

• Political resistance impacts potential OCV acceptability.
• Perceived vulnerability to cholera overrides rationale for OCV hesitancy.
• Case-by-case preemptive studies maximize public health intervention acceptability.

## 1 Introduction

**Fig. 1:**
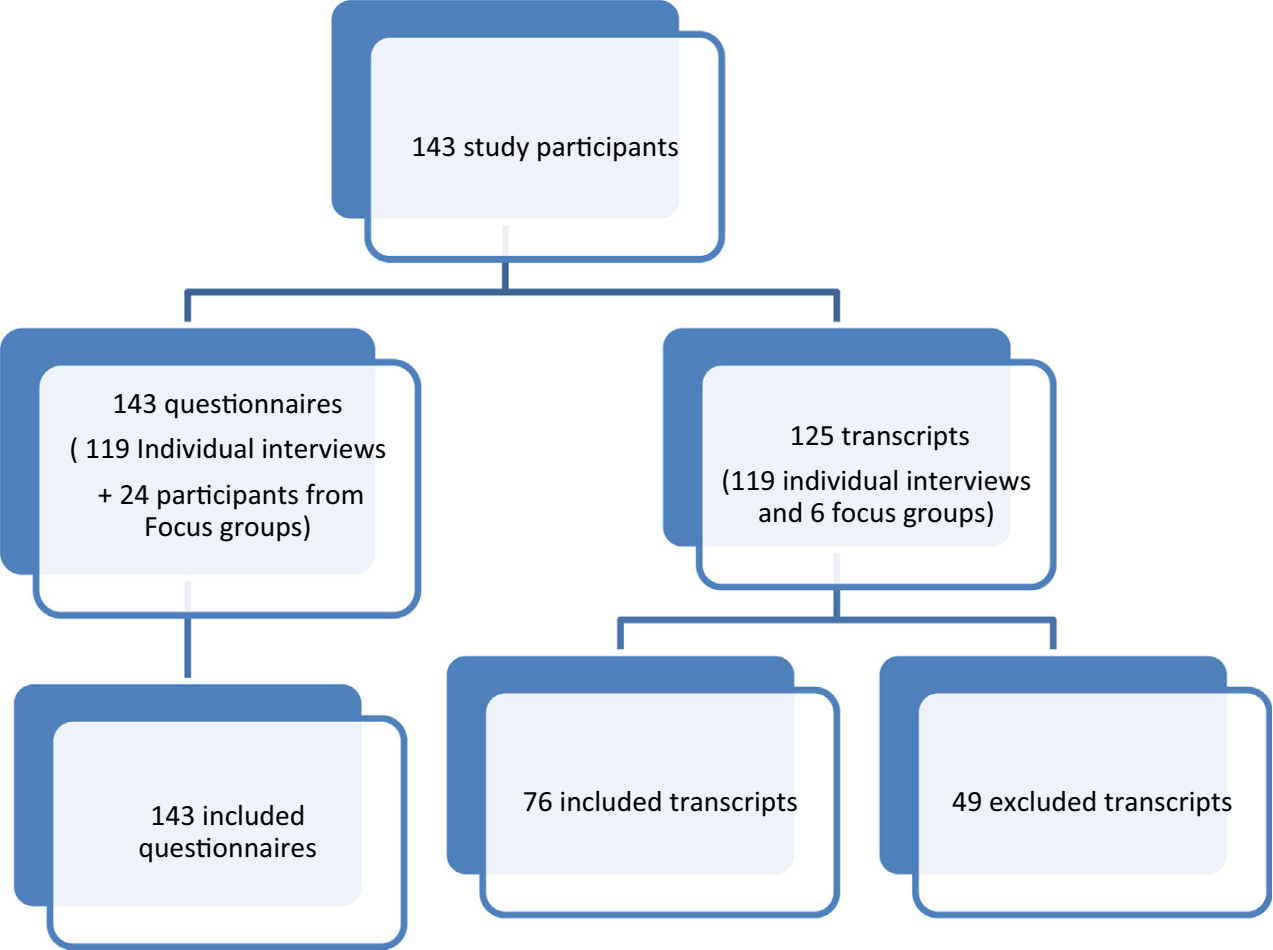
Flowchart of study participants included in the quantitative and qualitative analyses, pre-campaign assessment, June 2016, Nampula, Mozambique.

Mozambique has experienced several large cholera outbreaks over the past four decades [1] with 2536 cases reported between September 2015 and July 2016 alone. Half of all cholera cases (50.9%) were reported in Nampula City, the third largest city in Mozambique, situated in the north of the country [2] (Fig. 1). Two-thirds of these cases (66%) originated from six neighborhoods, which are characterized by poor sanitation (i.e., open defecation practices, poor waste collection, and degradation of the environment) and limited access to safe water.

The political context in Mozambique has changed over time, which may influence social determinants for the acceptability of health interventions. From 1976 to 1992, a civil war divided the country, with the Frelimo Government pitted against the opposition party, Renamo. Following a peace agreement and democratic elections during 1992, Frelimo continued to run the provincial and municipal governments in Nampula, until 2014. Currently, while the Mozambican Democratic Movement (MDM) runs the Nampula municipal government, Frelimo controls the national and provincial governments. It can be argued that politically motivated resistance impacts the acceptability of cholera preventive interventions.

In addition to curative interventions during cholera outbreaks, the country introduced preventive strategies in the 1980s, including campaigns for water chlorination, education, and information about cholera. In Nampula, episodes of violence occurred among the population during protests against water source-chlorination response teams. In 2009, 16 people were killed [3], [4] after having been accused of spreading cholera instead of preventing it [5].

Oral cholera vaccine (OCV) can prevent cholera in the short to medium term, while awaiting the longer term solution of improvements to water and sanitation infrastructures [6]. A pilot mass OCV immunization campaign was conducted in 2003 in Beira, using 40,000 doses [7]. In 2016, the Mozambique Ministry of Health (MoH) ordered 425,486 doses of Shanchol™ vaccine from the International Coordinating Group (ICG) – one of the three OCVs prequalified by the World Health Organization [8], [9] – to implement a two-dose OCV campaign in Nampula city.

Formative studies on vaccine acceptability, knowledge, and practices related to cholera are essential for the success of OCV campaigns [10], [11], [12]. Several studies also concluded on the importance of designing context-based strategies (for vaccine delivery, communication, and social mobilization) with the purpose of improving vaccine coverage [13] and preventing cholera more effectively [14].

We conducted a rapid anthropological assessment, in a community where resistance to cholera interventions has been reported, to evaluate potential barriers and levers for OCV acceptability, and to establish appropriate vaccination campaign and social mobilization strategies.

## 2 Methods

This rapid anthropological assessment was designed to investigate predefined topics (Table 1), using semi-structured interviews or focus groups and to allow the emergence of open (unexpected) answers based on the interviewees’ experiences.

**Table 1:** Key questions asked to community members for the qualitative assessment, pre-campaign assessment, June 2016, Nampula, Mozambique.

1. What is the name of your community and who is the chief here of this community? 2. Where do you get safe water here and do you have latrine? Do you have techniques to clean the water? 3. Have you ever heard about cholera? According to you, where does cholera come from? What do you know about cholera? How do you contract it? 4. Have you or someone you know ever had cholera? Could you describe what happened to her/him? 5. When confronted with severe diarrhea, what treatment would you use? 6. Have you experienced conflict on the issue of cholera in the past? 7. How do you feel about vaccines? If reluctant: Did you have a negative experience following a vaccination? 8. Is it easier for you do go to the local health facility or do you prefer it when vaccination teams come into your neighborhood? 9. Would you accept to be vaccinated against cholera? Would you accept to get your children vaccinated? 10. Do you prefer oral vaccine or injectable vaccine? (How do you feel about oral cholera vaccine? Do you think it is efficient? Do you think it is safe? Do you have fears concerning oral cholera vaccine? Do you think everyone should receive oral cholera vaccine?) 11. Who do you think should administer the vaccine: a professional from the health center, or someone from your community who has been trained to perform it? 12. How do you generally learn about health issues? Who would be the person, in your community, you would trust the most if they were relaying information about health behavior?

In addition, all study participants were asked to fill in short close-ended questionnaires to obtain quantitative data on circumscribed topics such as cholera experience and vaccine acceptability.

In-depth interviews and focus groups were tape-recorded when possible, and then transcribed. We excluded interviews if: (1) we were not allowed to record the interview and our notes were insufficient to produce comprehensive information, (2) the recorded sound quality was poor or (3) interviews were too short (less than 15 min) with no clear answers from the interviewees (Fig. 1, Flowchart of Study participants included in the quantitative and qualitative analyses). Qualitative data were analyzed using NVivo Software (Version 11) to perform thematic coding.

Questionnaires were anonymized and entered using the “Open Data Kit” platform. Data analyses were performed using the R software (R Project for Statistical Computing - Version 3.3.1). Quantitative results were derived from questionnaires to provide complementary information to support the interpretation of qualitative data. The sampling techniques used in this assessment were not designed to be representative of the study population and the results cannot be generalized to a broader population.

### 2.1 Study participants

**Fig. 2:**
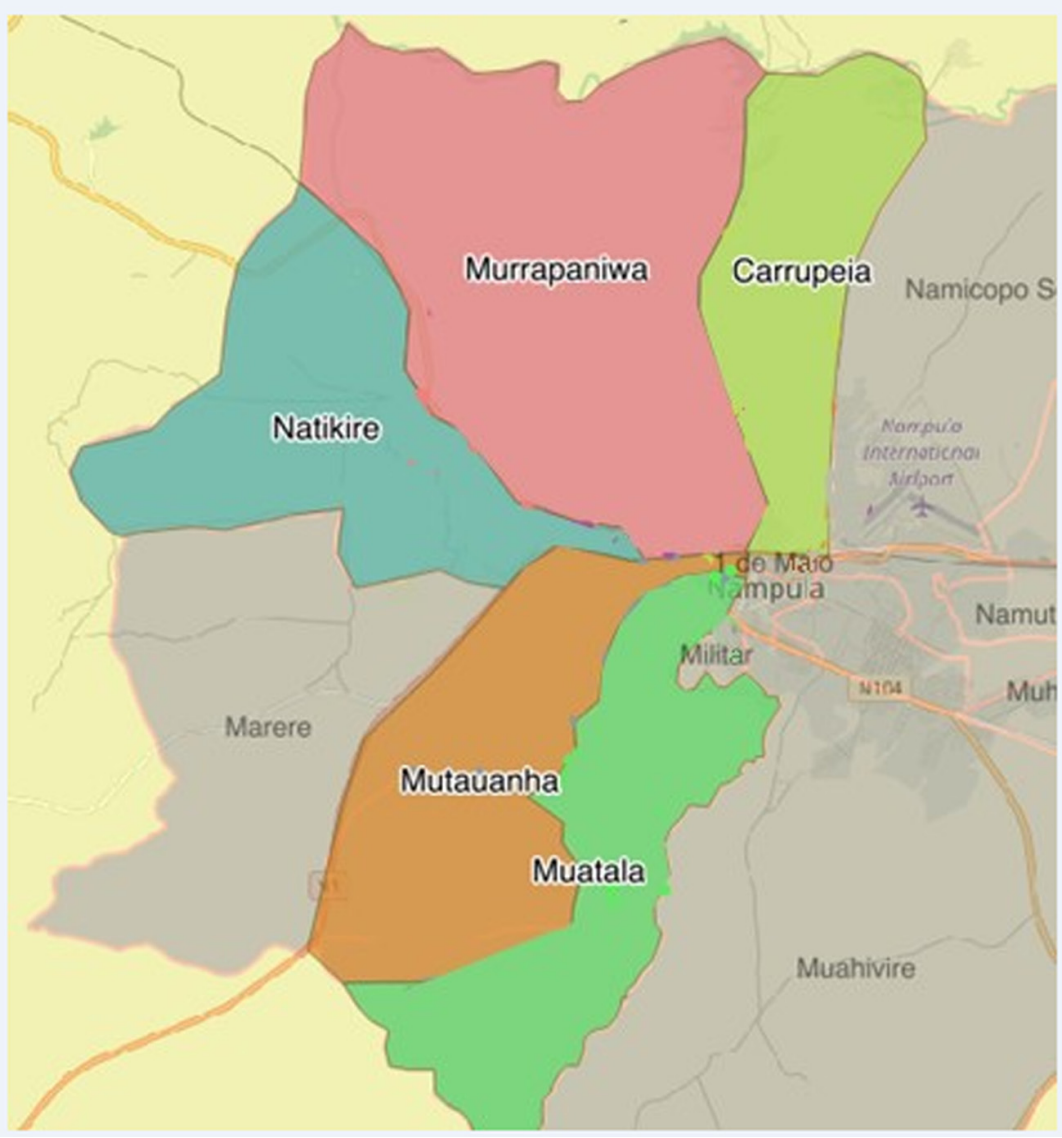
Map of the neighborhoods targeted by the OCV campaign, pre- campaign assessment, June 2016, Nampula, Mozambique.

Neighborhoods were selected for the OCV campaign from epidemiological records, based on higher cholera incidence between 2011 and 2016. Six of the 33 neighborhoods in Nampula City, cumulating 66% of the total number of cholera cases reported in Nampula City, were selected for the OCV campaign. Study participants were selected in three of these six neighborhoods that were most affected by cholera (Mutauanhana, Murrapaniwa and Muatala districts) (Fig. 2). We interviewed 143 persons, either through individual interviews (N = 119) or during the six focus group sessions (N = 24) (Table 2).

**Table 2:** Categories of total study participants, pre campaign assessment, June 2016, Nampula, Mozambique.

Cat Categories of participants	City of Nampula	Muatala	Mutauanha	TOT Murrapaniua	Total
Province representatives in Nampula	5				5
Political leaders at local level (municipality)	3				3
Other community representatives		4	4	8	16
Health workers	12				12
Community health volunteers	1	3	3	4	11
Community members (including those who have experienced cholera)		12	32	52	96

Total					143

For the qualitative analysis, a purposive, convenience and chain-referral sampling method was used. We selected key stakeholders at provincial and municipality levels (Table 2). At neighborhood, sub-neighborhood and unit levels, political representatives were selected as well as other community representatives, health workers, and community health volunteers. At the community level, we used purposive sampling to identify community members that met at least one of the three following selection criteria: (1) having personally experienced cholera, (2) having witnessed cholera episodes in their immediate surroundings (family, neighbors or friends) or (3) stating that they had a general knowledge of cholera. We identified study participants using chain-referral sampling [15]. Community leaders or unit chiefs assisted with identifying families who had experienced cholera during the last epidemics. At all levels, purposive sampling was based on referral by previous participants and convenience (potential respondent presence, availability and willingness to participate in the study) (see Table 3).

**Table 3:** Categories of Study Participants included in the Qualitative Analysis, pre-OCV campaign assessment, June 2016, Nampula, Mozambique.

Cat Categories of participants	City of Nampula	Muatala	Mutauanha	TOT Murrapaniua	Total
**Province representatives in Nampula** National Immunization Program Logistics Officer District health director Chief Public Health Officer Environmental Health and CTC disease prevention officer	4				4
**Political leaders at local level (municipality)** Municipality Hygiene Officer Municipality councilman for health and social affairs	2				2
Political leader at neighborhood level	2				2
Political leaders at the quarter level		1	1	1	3
Political leaders at the unit level		1	1	2	4
Other community representatives (community-based associations, religious leaders, teachers, informal leaders, etc.)		1	1	3	5
Health workers	6				6
Community health volunteers		3	4	4	11
Community members (including those who have experienced cholera)		11	11	15	37
Focus Group	2				2

Total	16	17	18	25	76

### 2.2 Ethical aspects

The CIBS-INS (*Comité Institutional de Bioetica do* Instituto Nnational de Saude [INS]) of Mozambique approved the protocol on January 4, 2016 (reference number 015/CIBS-INS2016). Additional authorizations were provided at each administrative level (Provincial Health Directorate, municipality, and community leaders for each sub-neighborhood). Participants provided voluntary, informed consent before participating in interviews.

## 3 Results

### 3.1 Perceptions of the origins of cholera

Among the study participants, 95% reported knowledge about what cholera is. When asked about the principal causes of cholera, 66% reported “dirtiness” (dirty water: 57% and dirty food: 39%). In-depth interviews provided information on alternative perceptions of what causes cholera. Some participants contrasted natural causes (lack of hygiene, transmission via oral-fecal routes) and unnatural causes and identified cholera as a “non-regular disease”, which was correlated with mistrust towards the government, whom respondents perceived to be responsible for cholera spread, motivated by political malice or negligence.

One narrative attributed the voluntary spreading of cholera to the government or its local representatives (Table 4). This occurred because local government representatives were believed to have been actively involved in paying unidentified third parties to pollute wells at night with cholera or because they authorized health workers to perform water chlorination (Table 4). A second narrative attributed pollution of wells to health workers, “people from the health area”. A third narrative indicated that representatives from the opposition, “MDM secretaries” may also be held responsible. Although the perpetrator designation narrative varied, a commonality was the differentiation between “important people” as opposed to “the people”. *“Since the disease only strikes the people and not the important people, they believe that there’s something going on”* (Community Health Volunteer).

**Table 4:** Quotations obtained from study participants, pre-OCV campaign assessment, June 2016, Nampula, Mozambique.

	Quotations	Respondent type
**Well chlorination associated incidents and alleged perpetrators**	“Well I heard that in the districts there have been ruckus, deaths, even nurses’ houses were burnt, they invade the hospitals, they beat”	Community member, Mutauanha

The perpetrators spreading cholera were believed to be health workers or secretaries or the government	“there are many accusations and people still believe the leader is bringing cholera or the government or the secretary, etc”	Community Health Volunteer, Mutauanha

Secretaries (local political representatives) were believed to be responsible for the cholera spread	“Because for example in Lalawe the neighborhood secretary was killed because he was accused of having brought the problem but even after he died cholera continued to exist, you get it?” “They found that secretary and went to break his house with the population, then he ran away” R: “They played drums at night with gallons saying ‘the secretaries are putting us bad, they’re finishing us’, that was their song. (..) They were really drumming and making a protest. Protesting that us, the secretaries, are bringing cholera” “It was through that cholera. A neighbor of ours was really sick so they told the secretaries that they’re the ones who promote that cholera and they started hitting him”	Mutauanha Health Worker/Ex CTC Health worker Unit Chief- Murrapaniua Murrapaniua Delegate

MdM Secretaries were believed to responsible for the cholera spread	R: “They didn’t start hitting us but there are slurs, they say stupid things like ‘the shitty secretary from MDM who’s bringing cholera here’. We do our things and they won’t help”	Mutauanha religious leader

Health Workers were held responsible for the cholera spread	“They believe that perhaps the health personnel besides bringing prevention measures are bringing something else, the disease itself. When they bring the chloride to disinfect the water, the people start saying that they're bringing the disease and they start spreading that misinformation to others, they start to influence them to not accept those measures.”	Unit Chief- Murrapaniua
	“Field health workers get cursed at, people say they bring cholera”(…) R: “The problem was that there were always muddles because they say ‘you’re the ones distributing it’, they always say ‘haaaa yes you’re distributing it’”	
	“Health staff arrives there they start saying ‘haaaa they’re bringing cholera’ and other stuff. We even brought this case to the police and the head of the department said it was us.”	Unit Chief- Murrapaniua

Local government representatives or “the government” were believed to pay third parties to pollute wells	“They said that the secretary would pay money and at night put it in the wells so that when people would get water from there, when they would drink it they’d get sick.” “Because someone, to understand cholera is (…) And in town, someone think about the cholera is something, for example, the government, if someone pay to go to put in some place, someone to go there to die”	Community Health Volunteers Center for Diarrheal Diseases- Nampula City – Responsible for Public Health and Communication

Head of Unit believed to be responsible because he authorized the health brigade to perform water chlorination	“Other people make a bad interpretation and say that it happens because it’s distributed. Even I, the Head of Unit, was accused of distributing cholera because I received a health brigade that was placing chlorine in wells and after we left we were beat up because they said the brigade wasn’t treating the water and were only spreading cholera so we weren’t being good.”	Head of Unit, Murrapaniua

Cholera strikes “the people” and not “the important people”	R: “A lot of the times the ones who suffer in those situations are the responsible for the neighborhoods…” P: “We’re talking about the heads of blocks or neighborhoods?” R: “Secretaries, heads of blocks, chiefs.” P: “Why do they accuse you?” R: “Because these people are members of the government. Since the disease only strikes the people and not the important people, they believe that there’s something going on so that has consequences some times.”	Community Health Volunteer

People believe the “dust” they observe while putting chlorine in the wells is cholera	“We talk about things the way they are because of this bad perception between chlorine and cholera, people associated that the ones who use the chlorine treatment leave some dust and that dust is cholera, so they made this association and started being very aggressive.”	Repartition Chief

The spread of cholera is performed at night by unknown perpetrators from the “health area”	“They said a man at night went to a house and when he got there he was preventing cholera and they started spreading it, so they were suspecting and they didn’t know who he was, they only knew he came because of cholera so they don’t want no one from the health area.”	Murrapaniua Delegate

The population and community leaders were not consulted nor informed before the intervention	“P: “OK and where do you think this misunderstanding came from? Why does the population have this misunderstanding about the source of cholera?” R: “Lack of information because I think the information shouldn’t be buried, if it wasn’t it would reduce the muddles. For example, in other neighborhoods they can get the population to obey to those people, they’re not like those troublemakers like Murrapaniua”.	Unit Chief- Murrapaniua

**Lack of public services are believed to be triggering cholera spread (i.e., lack of improved sanitation, and poor road development and waste management, lack of trash removal)**		

The lack of trash removal and improper sewage and water systems implies the population feels “forgotten”	“The way cholera is located (…) they feel they are forgotten, because they have no services arriving in this neighborhood; there’s no water, there is nothing, so they feel that they are forgotten, they are not part of society as such”	Murrapaniua, Primary school teacher

The lack of trash removal is linked to the cholera spread. The trash is “killing us”.	“When we don’t go to a certain area to remove the trash and there’s cholera there, the population is mad, and I receive sad and angry complaints like “why aren’t you coming to remove the trash that’s killing us?”	Municipality Representative

The Muatala river is used as trash disposal	“In our area we do not have a trash bin and everyone keeps it in their house and takes the trash to Muatala. Q: Muatala where? A: This river, I think it passes by here”	Muatala Community Member

The wells drain water from the same place the sewage runs through	“Nampula is a city in which we have rivers that are not actually rivers (in quotation marks) because they are sewers that run through the neighborhoods ... it is through these same sewers or drains of those sewers in which people drill their wells and find that same water and consume without treating.”	Mutauanha Health Worker/Ex CTC Health worker

**Children fleeing Polio Eradication Campaign**	“Whenever the vaccination came, there was panic, all the students were shouting, the people were running away (…) they could even go out through the windows.”	Murrapaniua, Primary School Teacher

**Rumors regarding vaccines following a death a few days after immunization**	“One time there in the school in Africa, at the Primary School Muatala, I think it happened three years, they vaccinated a girl there, the girl went home with this arm here inflamed, when it inflames, it also begins to create fevers. They accompanied her to the hospital, arriving at the hospital the girl dies. At the day of the funeral, population there in the neighborhood only spoke about the school's vaccine “ah the people from the school are who killed my daughter, they have killed my daughter because of that vaccine she got at school, her arm all swollen when we took her to hospital. We did not arrive on time, she died.” So the people who heard about that began to prohibit the children from getting vaccinated, began to prohibit their children from going to take something there in the “hospital” at school, so it creates this thing, this contradiction.”	Community Health Volunteer, Muatala.

**OCV may be refused as it be may bring cholera**	“They may deny it, saying ‘I don't want to get vaccinated, this vaccine will only create cholera, it will only bring cholera’”	Community member, Muatala.
	“Sometimes this is connected to cultural issues, sometimes political issues and they believe that perhaps the health personnel besides bringing prevention measures are bringing something else, the disease itself.”	Provincial Logistic Officer
	“It’s possible that the others start denying it, saying ‘I don't want to come get vaccinated, this vaccine will only raise cholera, they only brought cholera’ because I too hear this type of things on other populations.”	Muatala Community Health Volunteer

Political representatives relay the idea that the intervention is part of a population reduction plan organized by the opposite party	“When the Ministry of Health launches this publicity of prevention, other politicians objects: they say that are coming on purpose to bring cholera”	Nampula community leaders’ representative

	R: “Yes, it can really be for political reasons that these divergences occur, especially in this period we're in, this period of political divergences between the political parties.”	EPI logistics Officer- Nampula City-

**Community representatives’ involvement in the social mobilization is essential in order to reduce risks of acts of retaliation and successful implementation**	“First, we have to talk with the leaders” “They always have to create a friendship with the person in charge. If they don’t do that, they might end up being stoned or pulverized.” “The important thing in order to not have violence from the people is talking with the people until you introduce the vaccine.” “The secretary leader informs other secretaries from other blocks then the heads of the blocks call people (…) For example, if the secretary enters my house and gives me the information and he doesn’t give the information to my neighbor, I will also share the information. I will say ‘the secretary went to my house and said that from day X to day X, they’ll bring this’”	Health Director- Nampula city Nurse, Mutauanha Community Member, Muatala Community Health Volunteer, Murrapaniua

**Social mobilization, “explaining”, is critical, using gatherings and interpersonal communication**	“For the campaign it is important to create awareness, sensitizing.” “Before introducing it to the population first we’ll do a lecture about the vaccine, explain it so people understand and explain what’s going on. From then on you can do it, even I’m doing it, you can start. So for the population to accept it this is the advice I give you” P: “And how should those people come? How should those people bring the message about cholera matters so that the Cossoro community can understand?” R: “Summoning the people, sitting in a place so we can talk.”	Health Director- Nampula city Unit Chief Murrapaniua Muatala community Health Volunteer

Successful communication includes speaking the local language and using simple terms	“When I go there to explain in Macua someone will believe Yes so because you say in their local language? Yes(….) The most important thing we are able to identify activity in the community to explain about this case, we are able to use language simple so they may understand very well.”	Center for Diarrheal Diseases, Responsible for Public Health and Communication

**Successful trust-building initiatives include public intake by credible leaders**		

The teachers contribute as they take the tablets in front of the students so they may believe there is no danger in taking the tablet	“The teachers themselves who go there, (…) he takes the tablet in front of the students and the students see him, as an example, my teacher has taken it, so there is no danger of taking it, so it really contributes and it helps them to be aware that there is no danger in swallowing the tablets”	Murrapaniua-Primary school teacher

Drinking water purified with certeza in front of the families	“So you drink the purified water in front of them?” “Yes I drink in front of the people… yes … to believe me.”	Center for Diarrheal Diseases, Responsible for Public Health and Communication

Some respondents also attributed the spread of cholera to political negligence, mentioning a lack of improved sanitation, and poor road development and waste management. To support this argument, respondents identified the uneven geographical cholera distribution, inducing a perception of being “forgotten” (Table 4): *“why aren’t you coming to remove the trash that’s killing us?”* (Municipality Representative).

### 3.2 Experiences of conflict linked to cholera interventions

The qualitative data provided several narratives describing violent actions perpetrated in association with issues related to cholera (well chlorination intervention, emergence of cholera cases). Some respondents reported that they did not witness any conflicts, while others recalled numerous episodes of attacks with varying degrees of severity (i.e., insults and injuries, sit-ins, destruction of hospitals, and the murder of public health agents). The principal targets of these aggressions were nurses, health workers, activists or Secretaries (neighborhood municipality representatives), who represented the targeted public institutions. Respondents also reported a lack of community engagement in cholera interventions, as community leaders were not asked for permission or appropriately informed about planned interventions.

### 3.3 Attitude towards vaccines

Most participants described vaccines in general (i.e., not specifically OCV) in positive terms with regard to safety and efficacy. Some, however, reported difficulties with school-based polio vaccine introduction: “*Whenever the vaccination came, there was panic, all the students were shouting, the people were running away (…) they could even go out through the windows.”* (Murrapaniwa, Primary School Teacher). This behavior was a consequence of parents and children *“not being informed”* (Head Community Leader) – or being insufficiently informed – about the intervention.

The death of a girl following a school-based immunization session organized in 2013 in Muatala also triggered negative rumors about vaccines: “*On the day of the funeral, the population (…) said: ‘They have killed my daughter because of that vaccine she got at school’”* (Community Health Volunteer, Muatala).

Twenty-six percent of respondents reported previous negative experiences associated with immunization, mainly minor adverse events including fever, swelling, children crying, pain, pus formation, and inflammation for two weeks.

Explanations for potential vaccine hesitancy included fear of pain, and lack of knowledge of the benefits and importance of the vaccine.

Participants also expressed hesitancy regarding the immunization method: 55% of participants stated that they would prefer injections and 40% stated a preference for oral delivery. Several reasons were mentioned by the respondents: (1) Oral vaccine may be perceived as less efficacious than injectable vaccines, as the former do not directly enter the bloodstream; (2) the concentration of vaccine may be lower with oral than with injectable vaccines; and (3) young children might spit out the oral vaccine.

Some respondents pointed out their reluctance to be treated like “guinea pigs” by receiving an unknown vaccine.

### 3.4 Anticipated attitude towards OCV

As with cholera, OCV delivery was associated with political issues. At the community level, a rhetoric similar to the well pollution discourse was employed, namely that OCV campaigns may be used by an unidentified enemy or a political opponent to cause cholera. “*They may deny it, saying ‘I don't want to get vaccinated; this vaccine will only create cholera’*” (Community member, Muatala). Additionally, respondents indicated that political representatives themselves may believe and relay the idea that OCV can cause cholera. Contrasting with these negative reports, some Public Health representatives argued that political divisions end when it comes to combating cholera: *“all parties unite in the fight against cholera”* (Nampula City Health Directorate).

### 3.5 High level of intent to be vaccinated

Despite trust issues related to health interventions, the government, and vaccines in particular, the willingness to be vaccinated was very high (95%). This may be explained by respondents reporting a high perceived vulnerability to cholera, i.e., their awareness of its severity and importance, describing it as a “serious”, “real”, and “deadly” disease. This vulnerability was fueled by the perception that insufficient resources existed to combat cholera. In addition, a majority of respondents reported having personally experienced or had a family member or friend who had experienced cholera.

### 3.6 Preferred immunization strategies

Respondents mostly preferred the vaccine to be administered by health professionals rather than community health workers (83% vs. 11%) and favored a public health center-based delivery rather than a home-based delivery (75% versus 6%). This is explained by the fact that (1) respondents perceive safe delivery to be ensured by the presence of health professionals, (2) their lack of experience with house-to-house immunization performed by health professionals, and (3) a concern that persons who are absent at the time of the visit would miss their opportunity. During interviews, respondents often suggested using an outreach strategy based in the community.

### 3.7 Communication strategies and successful local initiatives

Nampula public health officials indicated that they had led trust-building initiatives to overcome the issues associated with well chlorination. For example, the name of the substance used to purify the water was changed from “cloro” – which was perceived as resembling “cólera” – to “certeza”. They also performed house-to-house information campaigns, and social mobilizers demonstrated the safety of purified water by drinking it themselves in front of community members. Similarly, new medication introduction hesitancy was mitigated thanks to the involvement of primary school teachers, who took the tablets themselves in front of their pupils (Table 4).

In Nampula, recommended communication strategies included information provided by social mobilizers using a door-to-door strategy; messages delivered via a megaphone in churches, schools, mosques, markets, and in the streets; and campaign announcements on television and radio. Community leaders’ involvement (via interpersonal communication and collective gatherings), was described as critical for mitigating potential conflicts. Bypassing the authority of community representatives was reported to increase the risk of retaliatory acts (Table 4).

Respondents indicated that key messages should be conveyed in simple terms, using the local language, and should include all necessary information related to: (1) campaign logistics; (2) the safety of a vaccine delivered by the oral route; (3) details on past OCV implementation in Mozambique and other countries; (4) individual ability to refuse the vaccine.

## 4 Discussion

Politics, in some cases, have an important impact on vaccine acceptance. Acute resistance to polio eradication campaigns associated with distrust in the government and North-South political antagonism have been documented [16], [17], [18].

In Mozambique, strong resistance to IPTi (Intermittent Preventive Treatment in infants) trials has been observed as they were implemented by a government clinic in areas that were strongholds of the opposition party [19].

In Nampula, opposition to the central and provincial government party (Frelimo) has long been documented and may have led to resistance against previous immunization campaigns and water chlorination interventions as well as reported hesitancy towards OCV campaigns. In our study, rumors portraying a malevolent opposing party potentially using the campaign to spread cholera were relayed by political actors themselves: *“When the Ministry of Health launches this publicity of prevention, other politicians object: they say that they are coming with the purpose of bringing cholera*” (Nampula community leaders’ representative).

Corroborating Serra’s findings on the well chlorination riots [3], this case study reveals, however, that in Nampula, potential vaccine acceptance or refusal arises not only from political divisions; rather, it is a consequence of despair in the face of perceived social inequality, insecurity, and government inaction. Social inequality may trigger attitudes of OCV hesitancy and lack of trust in those perceived as “the rich and the powerful”, the “select few” who are thought to reap the benefits of the development of Nampula city [20], [3]. In our study, some Nampula residents distinguished between the “important people”, never affected by cholera, and the poor and vulnerable, “the people”, affected by the disease. Perceived insecurity (fear of being killed by an unidentified enemy or political opponent) likely exists because of the long-lasting civil war that pitted the coastal region against the central government, with some Nampula residents reporting a sense of still being at war, albeit in a different form [21]. Finally, vaccine refusals have been linked to immunization campaigns in contexts where demand refusal or inadequate funding for general health services and routine immunization is observed [22]. While Briggs and Briggs described cholera spread as a racially institutionalized uneven access to health services for the Warao [23], Nampula respondents pointed to an institutional negligence, the lack of public waste management services as a root cause of cholera spread, which then translated into the perception of being abandoned by the central government and subsequent distrust in a variety of government-provided health interventions.

Despite mistrust towards the government, the high level of intent to be vaccinated (95%) may indicate that personal experiences of cholera would override motives for hesitant behaviors as most respondents described a personal experience of cholera and displayed a high level of perceived vulnerability to the disease [24]. Risk perceptions are described as predictors of adult vaccination behavior, and consist of two components [27]: perceived severity of consequences and perceived vulnerability to disease. Perceived cholera severity may influence demand for enteric vaccines [25] and was also identified as a positive determinant of OCV uptake [24]. However, perceived cholera vulnerability has not been previously reported to affect OCV acceptance, despite an association between OCV acceptance and the psychological and personal impact of cholera [26]. Our study provides data correlating both dimensions of risk perception to anticipated OCV acceptance.

The anthropological approach enables the assessment of current perceptions and political dynamics, which orients the implementation of public health initiatives and enables the elaboration of collaborative community-based communication strategies and health interventions. While general principles exist, our findings illustrate the importance of understanding the local context in detail, prior to implementing immunization.

Our study had several limitations. A purposive sampling technique was used to select participants for the interviews; our findings might not be fully representative of the targeted population. Additionally, cultural, social, and historical determinants may vary geographically and temporally, so our results may not be representative of other populations or of the population of Nampula at a later time; this, however, is true of all anthropological studies of this nature and does not invalidate the general principles we found.

## 5 Recommendations

Despite reports that the routine immunization program in Mozambique is viewed favorably [19], [28], our study leads to several recommendations for campaigns and also potentially for routine immunization in some regions (the two previously cited studies were conducted in areas of Mozambique to the south of Nampula). Although Nampula public officials may assume that all political parties will work together to promote health, this may not necessarily occur and hence political antagonisms should be taken into consideration in the implementation of immunization campaigns. For example, it may be critical that representatives of different political parties at administrative and community levels be equally involved in social mobilization efforts, before and during campaigns. During campaigns, public health officials should promote other planned interventions (such as water, sanitation, and hygiene improvements) to mitigate the lack of trust associated with perceived institutional negligence. Finally, there is a role for locally based trust-building initiatives to promote community engagement. Successful past initiatives have included health workers demonstrating the safety of purified water by drinking it themselves, and public-distributed medication intake or vaccine receipt by social mobilizers, teachers or credible leaders.

## Authorship contribution

EG developed the study protocol and questionnaires with inputs from RD, CB, LH and PC. CB and RD supervised the data collection. RD, CB and CS performed the data collection. LH supervised the NVivo data analysis. Analysis and interpretation of the data was conducted by RD, LH, CS and AM. RD coordinated the draft report. RD and LH drafted the study report with inputs from CB, CS, PC and AM. PC, MM and JBLG provided guidance and oversight throughout the study: PC as project director, JBLG as Health Economics and Medical Anthropology Program Leader and MM as primary investigator of the “VaxiChol” project for OCV monitoring and evaluation within which the present study was nested. RD was the main author of the article with significant contribution from EG. BDG provided input into study design and extensively revised the first manuscript draft. All authors critically reviewed and have given final approval of the manuscript.
